# Coordinative Motion-Based Bilateral Rehabilitation Training System with Exoskeleton and Haptic Devices for Biomedical Application

**DOI:** 10.3390/mi10010008

**Published:** 2018-12-24

**Authors:** Songyuan Zhang, Qiang Fu, Shuxiang Guo, Yili Fu

**Affiliations:** 1 State Key Laboratory of Robotics and System, Harbin Institute of Technology, Harbin 150001, China; meylfu@hit.edu.cn; 2 Tianjin Key Laboratory for Control Theory & Application in Complicated Systems and Biomedical Robot Laboratory, School of Electrical and Electronic Engineering, Tianjin University of Technology, Binshui Xidao 391, Tianjin 300384, China; 3 Department of Intelligent Mechanical Systems Engineering, Kagawa University, 2217-20 Hayashi-cho, Takamatsu 761-0396, Japan; guo@eng.kagawa-u.ac.jp

**Keywords:** robot-assisted, bilateral training, upper limb, exoskeleton device, tactile feedback, visual feedback

## Abstract

According to the neuro-rehabilitation theory, compared with unilateral training, bilateral training is proven to be an effective method for hemiparesis, which affects the most part of stroke patients. In this study, a novel bilateral rehabilitation training system, which incorporates a lightweight exoskeleton device worn on the affected limb; a haptic device (Phantom Premium), which is used for generating a desired tactile feedback for the affected limb; and a VR (virtual reality) graphic interface, has been developed. The use of VR technology during rehabilitation can provide goal directed tasks with rewards and motivate the patient to undertake extended rehabilitation. This paper is mainly focused on elbow joint training, and other independent joints can be trained by easily changing the VR training interface. The haptic device is adopted to enable patients to use their own decision making abilities with a tactical feedback. Integrated with a VR-based graphic interface, the goal-oriented task can help to gradually recovery their motor function with a coordinative motion between two limbs. In particular, the proposed system can accelerate neural plasticity and motor recovery in those patients with little muscle strength by using the exoskeleton device. The exoskeleton device can provide from relatively high joint impedance to near-zero impedance, and can provide a partial assist as the patient requires.

## 1. Introduction

In the United States, 795,000 new or recurrent strokes are reported every year; many of these stroke victims suffer from motor impairment [1]. Considering that, up to two-thirds of stroke survivors suffer from upper limb dysfunctions and 80% of stroke survivors refer to a one-sided weakness called hemiparesis [2]. Studies have proven the plasticity of humans’ neurons, and that individual motor experiences can alter motor cortex functions [3]. Rehabilitation training can shape subsequent reorganization in the adjacent intact cortex after local damage to the motor cortex, and the undamaged motor cortex may play an important role in motor recovery. This is a precondition of physical rehabilitation; some training strategies that are beneficial in recovery from strokes have been reported, including unilateral training [4,5] and bilateral training [6,7,8]. Among them, bilateral arm training has shown promise in expediting progress toward upper limb recovery for stroke patients with hemiparesis. 

For delivering the burden of therapists for the ageing society, robot-mediated therapists for stroke survivors were adopted. Robot-mediated training can be traced back to 1990s; since then, many robotic devices have become commercially available in clinics and hospitals [9,10,11]. Previous studies proved that the achievement of the goal-oriented task can be facilitated with feedback, which can provide information on errors in movement and direction to correct them by speeding up the learning process or increasing the level of motor skill attained [12]. Several studies have focused on robot-assisted bilateral upper limb training for motor function recovery of activities of daily living (ADL). Furthermore, virtual reality technology has commonly been applied to bilateral training for increasing the motivation of patients [13,14], such as the EXO-UL7 exoskeleton robot (Bionics Lab, Los Angeles, CA, USA), designed with two arms with each arm having 7 DoFs. When bilateral training is performed, the desired joint angles are symmetrically transmitted from the less impaired upper limb to the impaired upper limb [15]. Miao Q. et al. designed a training system in which two robotic devices that work cooperatively with patients were involved, and in which appropriate trajectories can be determined for robot-assisted bilateral upper limb training with a novel three-stage trajectory generation method [16]. Song Z. et al. designed a novel upper extremity motor function rehabilitation system. Patients can perform a tracking task in a virtual environment with coordination training of bilateral upper extremity through a haptic device and an inertia sensor [17]. Our previous research proposed an upper limb elbow joint representation method that uses only a one-channel electromyography (EMG) signal implemented for bilateral neuro-rehabilitation [18,19].

**Figure 1:**
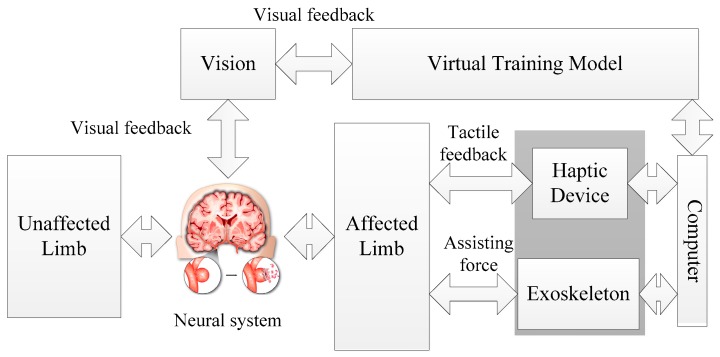
Neuro-training with the proposed system.

In this paper, a novel bilateral rehabilitation training system with coordinative motion of two limbs is proposed. The principle of the neuro-training with the proposed system is shown in Figure 1. Both affected and unaffected limbs perform the same movements simultaneously and independently of each other according to the virtual training model. Tactile feedback as well as visual feedback can provide information on errors in movement during task-oriented training. The exoskeleton used in this system is a power assisting device for providing an assist-as-needed force. By combining the haptic device with a power assisting device, the training system can perform training for patients with different motor functions.

The contribution of our designed system with respect to the existing bilateral rehabilitation training system is summarized as follows: Firstly, the proposed system integrated an exoskeleton device for providing assistive or resistive force and a haptic device that can provide “haptic guidance”, which helps patients to be aware of the error between affected and unaffected limbs. For the assistive force provided by the exoskeleton, the robot will guide patients while assisting them toward the correct path for patients that cannot execute the required task. For resistive force, the exoskeleton may dissipate partial energy from patients, which may increase the task difficulties. Therefore, the system can be used for a wide range of patients with different motor abilities. Secondly, for most bilateral systems, the symmetric devices on both sides are applied and bilateral symmetrical movements are allowed. In contrast, for the proposed system, an inertia sensor is used for detecting the motion of the unaffected limb, and it performs the coordinative motion with the affected limb in order to activate similar neural networks in both hemispheres. The inertia sensors used can reduce the cost of total system without restricting the motion of the unaffected limb. This method also has good expansibility, that is, the orientation of the patient’s upper limb can be provided with four inertial sensors mounted in special places for further training on the whole forearm.

The remainder of this paper is organized as follows. In Section 2, the system overview is given. In particular, the exoskeleton device design and its impedance generation principle are introduced. In Section 3, the proposed bilateral training model and the tactical feedback related to the training model are introduced. In Section 4, the bilateral training, which is used for elbow function recovery, is introduced and several experiments involving three volunteers are carried out. In Section 5, the results and discussion are given. The performance of the proposed system is evaluated with the mean squared error further between the affected and unaffected arm for each trial. Finally, the conclusion and future works are discussed.

## 2. System Overview

**Figure 2:**
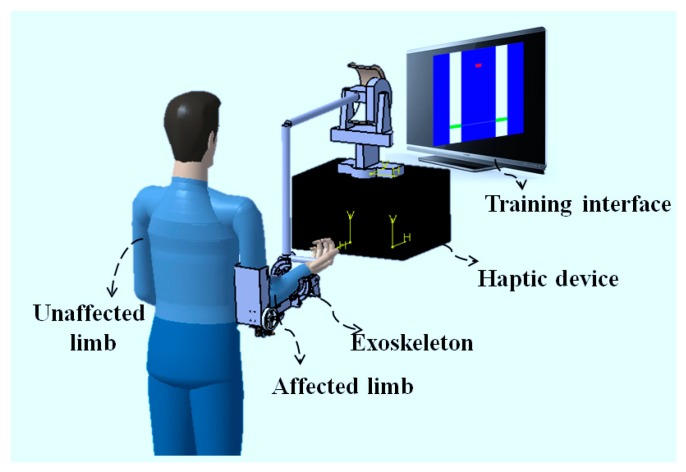
Bilateral rehabilitation training system.

**Figure 3:**
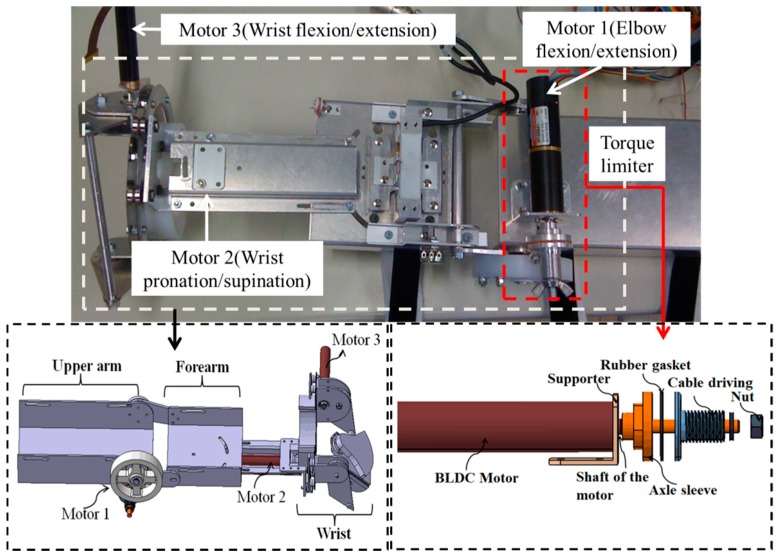
The exoskeleton device (lower view).

Figure 2 shows the conceptual design of the bilateral training system for recovering the motor function of the elbow joint, including flexion and extension motions. The hardware of the training system consists of a commercial haptic device (Phantom Premium 3.0, 3D Systems, Rock Hill, SC, USA), a lightweight exoskeleton, and a computer combined with a graphic interface. The Phantom Premium is superior to the Omni device previously used on several aspects, including the workspace, the tolerated torque, and so on. The Phantom Premium is also easy to operate and set a desired torque in the horizontal and vertical planes. The exoskeleton device can provide an assist-as-needed force during the training. The training interface is developed with Microsoft Visual Studio 2010 and the virtual reality is rendered by Open Graphics Library (OpenGL, Khronos Group, Beaverton, OR, USA), as shown in Figure 3. Multithreading is adopted for recording the data, generating the tactile feedback with the haptic device, and providing the power assisting synchronously. In the provided program, the speed of the Phantom thread is faster than that of inertia sensors for guaranteeing tactile feedback transparency. The graphic interface can also show the actual angle of the two limbs, with which the subject can know their condition immediately. Therefore, both visual and tactile feedback can be provided to the patient, while both affected and unaffected limbs perform the same movements simultaneously. The training data can also be recorded for further assessment by the therapist. Therapists can know the recovery status of patients, as well as make further treatment prescriptions. The patient can thus retain the experience of motion in the unaffected limb, and the motor function of the affected limb can recover under the guidance of this experience, especially for hemiparesis stroke patients.

In the following subsections, the exoskeleton device design and its impedance generation principle, providing from relatively high joint impedance to near-zero impedance, will be introduced. In particular, a safety consideration with a clutch-like mechanism is used. During bilateral training, the motion of the unaffected limb is detected with the MTx sensor (Xsens, Enschede, The Netherlands), while the motion of the affected limb is calculated from the coordinate transformation of the haptic device, which will be introduced below.

### 2.1. Exoskeleton Device Design

Compared with the unaffected limb, the affected limb lacks motor function, which results in less control of the affected limb. The exoskeleton device is worn by the affected limb for providing partial power assistance, which is decided by the needs of the patient. The signal can also come from the biological signal, where the surface electromyography (sEMG) can record the activation level of skeleton muscles, which is a more accurate method for determining the amount of force exerted [20,21,22].

The exoskeleton device previously designed in our group allows an ergonomic physical human–robot interface, as shown in Figure 3, which realizes a convenient wearing and comfortable interaction [23,24,25]. This device can be easily worn by the caregiver or the patient. Constant alignment between the user’s elbow and exoskeleton axes during movement is realized with two passive DoFs mechanisms, which can avoid the translational forces caused by joint misalignment. The power derived from the motor is transmitted to a drive pulley through a stainless steel wire rope in the actuated DoFs of the elbow joint, which not only provides enough torque, but also decreases the backlash usually observed in geared mechanisms. The exoskeleton device has enough power (max. continuous torque: 14.2 Nm) to realize the desired positioning performance. For the active assistance, instead of using the traditional impedance control and admittance control schemes, a control method relating to the series elastic actuator (SEA) was used [26,27]. This method decreases the inertia and intrinsic impedance of the actuator, which allows more accurate and stable force control.

### 2.2. Impedance Generation Principle with Exoskeleton Device

The force bandwidth of the exoskeleton device determines how much power the device assists or resists patients. The detailed information for controlling the exoskeleton device with an extended impedance method is illustrated below [28].

**Figure 4:**
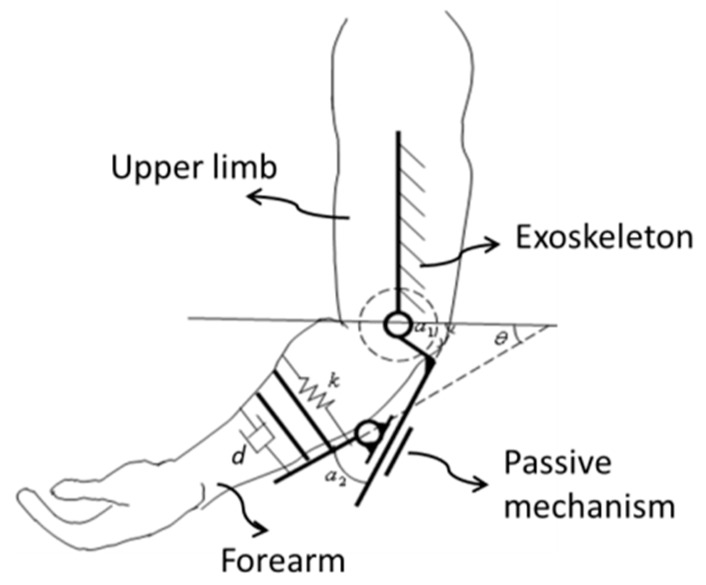
Schematic of the exoskeleton.

Figure 4 shows the mechanism schematic of the exoskeleton device on the elbow joint. While the patient moves his/her limb in constant velocity, the force exerted on the affected limb can be calculated by the mass–spring–damper model, which is given by Equation (1).

(1) $$F-d{\dot{\theta }}_{1}=k\left(\theta_{1}-\theta \right)$$

where *F* is the virtual assistive or resistive force to patients when the patient moves his arm in constant velocity, *k* is the spring coefficient, and *d* is the damping coefficient. We can adjust the *k* and *d* to set in different virtual environments. *θ*_1_ is the angle between the affected limb and horizontal plane, which is measured with the haptic device and is not shown in Figure 4; and *θ* is the angle between forearm frame and horizontal plane. 

According the mechanism of the elbow joint, the rotational angle of the device α_1_ can be obtained by Equation (2).

(2) $$\alpha_{1}=\alpha_{2}^{}+\theta$$

where *α*_2_ is the angle of the passive rotational joint, which can be detected by a potentiometer.

Then, the velocity relationship can be obtained by Equation (3).

(3) $$\theta =\theta_{1}+\frac{1}{k}\left(d{\dot{\theta }}_{1}-F\right)$$

Here, we assume the assisting or resisting force is constant, and the rotational velocity of the affected limb is calculated by Equation (4).

(4) $${\dot{\alpha }}_{1}={\dot{\alpha }}_{2}+{\dot{\theta }}_{1}+\frac{1}{k}\left(d{\ddot{\theta }}_{1}-\dot{F}\right)$$

Here, the angles of the upper limb motion can be determined via integration of kinematics of one upper limb and the haptic device [28]. It should be noted that in order to obtain a relatively accurate value, the motor pattern of the upper limb must be fixed, otherwise some parameter may vary while calculating the forward kinematics.

### 2.3. Safety Considerations During Training with the Exoskeleton Device

In our study, force assisting with the exoskeleton is focused on those patients with little motor function. To avoid any danger in the absence of professionally supervised assistance, a torque-limiter mechanism installed on the exoskeleton is proposed for ensuring the patients’ safety, as shown in Figure 3 [24]. For the torque-limiter mechanism, the axle sleeve is fixed to the shaft of motor. The cable driving part is connected to the axle sleeve with a rubber gasket, which can provide the fiction force by tightening the nut. The compression force between the axle sleeve and cable driving part can be adjusted with a nut. When the external torque to the cable driving part is less than the preset threshold, the motor can drive the device to move, that is, the cable driving part can rotate together with the axle sleeve. If the external torque is larger than the threshold, the cable driving part and axle sleeve part will not rotate together anymore. Therefore, the safety of patients can be guaranteed because the cable driving part will rotate independently of the motor. 

### 2.4. Motion Detection of the Affected Limb with Haptic Devices

**Figure 5:**
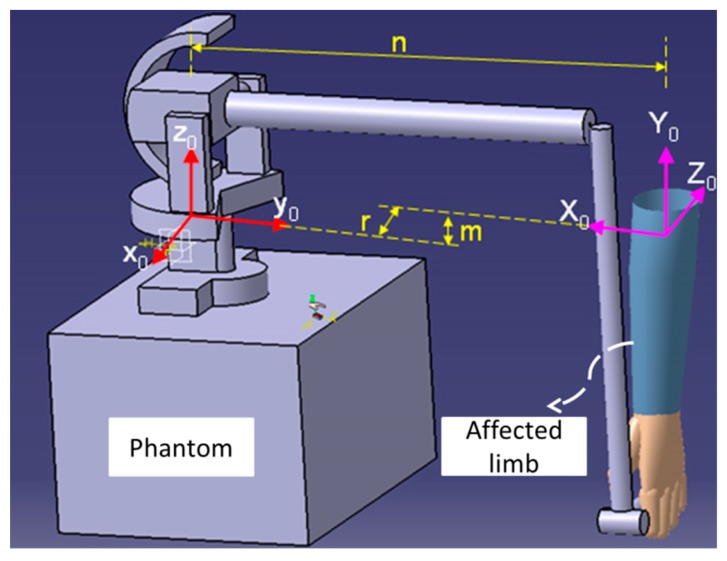
Frame assignment for the upper limb and the Phantom Premium.

During the bilateral rehabilitation training, the user grips the end-effector of the Phantom Premium with their hand, as shown in Figure 5. The position and posture of the end-effector of the Phantom Premium are calculated by forward kinematics, which is calculated by Denavit–Hartenberg (DH) algorithms. The transformation matrix from the original frame of the upper limb to the Phantom Premium is given by Equations (5) and (6), and the elbow extension/flexion motion of patients is calculated by Equation (7).

(5) AhP=[00−1−r−100n010m0001]

(6) T04=AhPA04

(7) $$\theta_{1}=\arccos\frac{s_{1}c_{2+3}s_{5}-s_{1}s_{2+3}s_{4}c_{5}+c_{1}c_{4}c_{5}}{s\arccos(-c_{1}c_{2+3}s_{5}+c_{1}s_{2+3}s_{4}c_{5}+s_{1}c_{4}c_{5})}$$

where *n*, *r*, and *m* are the displacements between original coordination of the Phantom Premium and upper limb along the *x*, *y*, and *z* axes, respectively. *θ*_1_ is the angle of the elbow extension and flexion, *c* stands for cosine, and *s* stands for sine [28].

## 3. Methods and Materials

A bilateral training model on the sagittal plane for elbow joint recovery is proposed. The motor function can be recovered with a cooperative training between the affected limb and unaffected limb via both force and virtual feedback. An open-loop impedance controller for haptic device is introduced here, which is related to the training model. In particular, the desired environmental impedance can be adjusted for different trainings. 

### 3.1. Bilateral Training Model for Hemiparesis on the Elbow Joint

**Figure 6:**
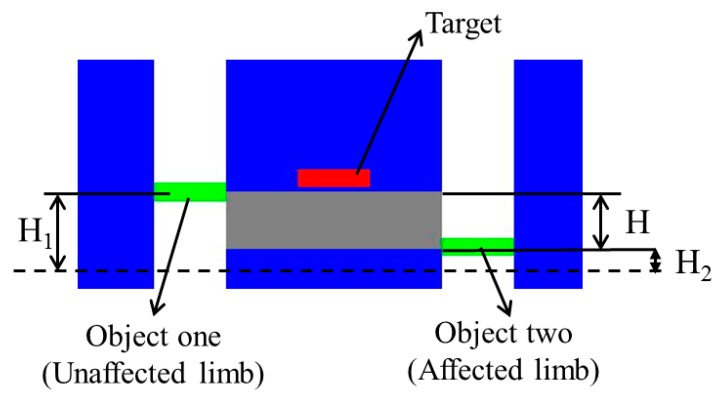
Proposed training model.

The training model is built according to the neuro-rehabilitation theory, as shown in Figure 6. Training for motor function recovery can retrain the neural function, which could result in enhancement of representational plasticity and functional motor recovery. Both the affected and unaffected limbs should start from an initial position where two limbs vertically relax. Then, the affected arm wearing an exoskeleton device will operate the handle of the Phantom while synchronously moving together with the unaffected limb to the target position. The position of the target can randomly change along a vertical line within a defined range after some limitations are satisfied. *H*_1_ and *H*_2_ represent the height showed in graphic interface; the height *H*_1_ is relative to the flexion/extension angle (Euler angle) recorded with an inertia sensor (MTx sensor, Xsens, Enschede, the Netherlands) [29], and *H*_2_ is calculated from the relative kinematics with the Phantom [21]. Here, the inertial sensor used for detecting the motion of the unaffected limb can get the gesture of the upper limb in real-time when it is equipped on the patient’s limb [30]. It can provide drift-free 3D orientation as well as kinematic data; namely, 3D acceleration, 3D rate of turn (rate gyro), and 3D Earth-magnetic field. The relationship between the coordinates of the inertia sensor and the reference coordinates can be calculated by a rotation matrix *R*. The rotation matrix, which contains information about the relative position of the two coordinate systems, is defined by Equation (8).

(8) $$R=R_{\psi }^{Z}R_{\theta }^{Y}R_{\varphi }^{X}$$

Here,

(9) $$R_{\psi }^{Z}=\left[\begin{array}{ccc} \cos\psi & -\sin\psi & 0\\ \sin\psi & \cos\psi & 0\\ 0 & 0 & 1 \end{array}\right]$$

(10) $$R_{\theta }^{Y}=\left[\begin{array}{ccc} \cos\theta_{2} & 0 & \sin\theta_{2}\\ 0 & 1 & 0\\ -\sin\theta_{2} & 0 & \cos\theta_{2} \end{array}\right]$$

(11) $$R_{\varphi }^{X}=\left[\begin{array}{ccc} 1 & 0 & 0\\ 0 & \cos\varphi & -\sin\varphi \\ 0 & \sin\varphi & \cos\varphi \end{array}\right]$$

where *ψ* represents the rotation about the *z*-axis (yaw), *θ*_2_ represents the rotation about the *y*-axis (pitch), and *φ* represents the rotation about the *x*-axis (roll). 

During the bilateral training, the target changes its position once the gray area, which shows the difference between the two limbs, overlaps the target, as well as when Equation (12) is satisfied.

(12) $$\left|H_{1}-H_{2}\right|=H$$

where *H* represents the difference angle detected between the affected and unaffected limbs.

Considering that every patient has a different physical ability and condition, the value of the threshold *H* can be adjusted according to the training difficulty determined by the stroke degree of patients. Here, the threshold is determined by the flow theory [31] with which patients can achieve an optimal training experience to keep the balance between training difficulty level and patient status. In our previous research, a muscle strength assessment system, which records the activation level of skeleton muscles, was proposed [19], with which the patient can be trained within the range of optimal training, where the difficulty level of training is in accordance with his/her muscle strength. When the training difficulty level exceeds patients’ ability, overtraining will happen; on the contrary, undertraining will happen when the opposite occurs.

### 3.2. Tactile Feedback with Haptic Device

**Figure 7:**
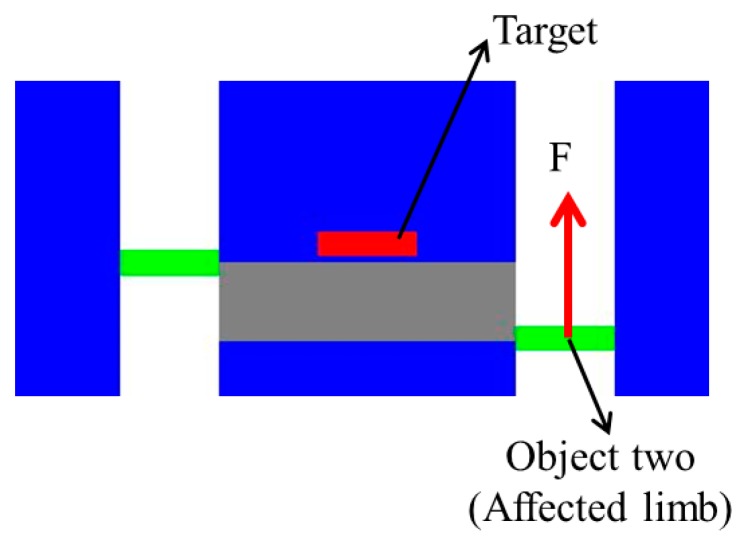
Upward feedback provided by the haptic device.

As mentioned above, the haptic device can generate the necessary tactile feedback, thus the patient can determine their motion and correct the error between the two limbs according to the force magnitude and direction. In particular, the affected limb can receive the feedback with two directions; if the affected limb is located above the unaffected limb, a force downward is provided, while on the contrary, an upward force is provided when the opposite occurs, as shown in Figure 7. The magnitude of feedback is relative to the error, thus the received feedback can provide information on errors in movement.

**Figure 8:**
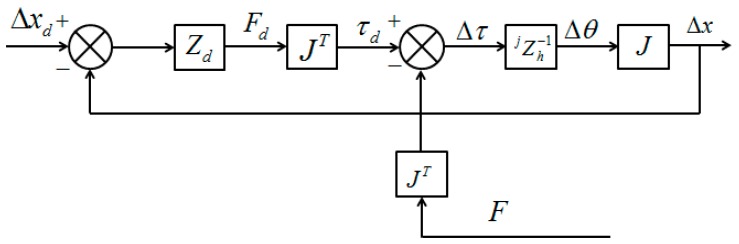
Open-loop impedance controller for the Phantom Premium.

As shown in Figure 7, where the affected limb is located under the unaffected limb, an upward tactile feedback is provided. The open-loop impedance controller used for the haptic device is illustrated in Figure 8 [32]. In this controller, $Z_{h}^{-1}$ represents the linearized device dynamics between the torque input $\tau_{d}$ and the differential angular output Δθ, and *J* represents the relationship between the Cartesian position of haptic device’s handle and the joint position of the device. The desired position deflection is shown in Equation (13).

(13) $$\Delta x_{d}=x_{d}-x_{0}$$

The actual deflection is shown in Equation (14).

(14) $$\Delta x=x-x_{0}$$

Therefore, the feedback command *F_d_* can be obtained with Equation (15).

(15) $$F_{d}=Z_{d}(\Delta x_{d}-\Delta x)=Z_{d}\left|H\right|$$

In the training model, the desired environmental impedance *Z_d_* is set as a spring condition for obtaining information on errors in movement and the elastic coefficient is selected with the force sensing ability of humans and the tolerated torque of the haptic device. The environmental impedance can also be adjusted following task requirement for goal directed tasks with rewards.

## 4. Experiments for Bilateral Rehabilitation Training

Measuring the muscle activity is a more accurate method for determining the amount of force exerted, which can be measured with surface electromyography (sEMG). In this section, tactile feedback with the haptic device is evaluated with experiments that compare exerted force and sEMG signals. Moreover, the performance evaluation of the bilateral training system is carried out. 

### 4.1. Performance Evaluation of the Tactile Feedback with the Haptic Device

**Figure 9:**
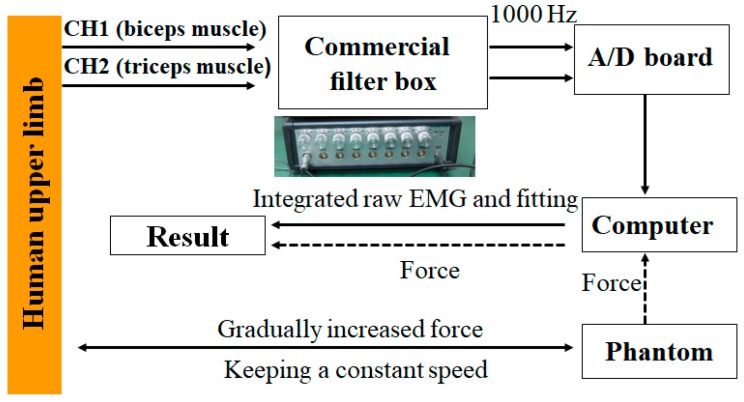
Data recording and processing process. EMG—electromyography; A/D—analog to digital.

**Figure 10:**
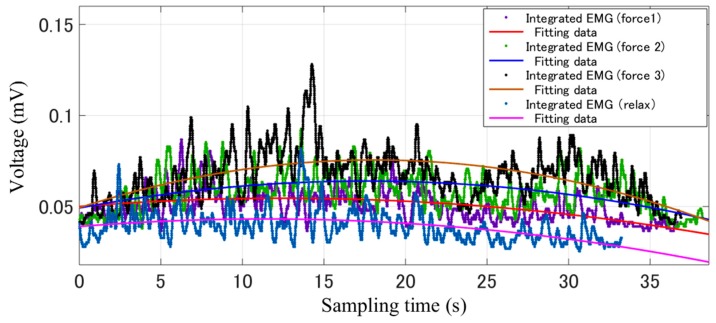
Integrated EMG signals and fitting data.

Figure 9 shows the experimental process for verifying the tactile feedback with the haptic device. After recording the sEMG signals from the bicep and tricep muscles while the haptic device exerts a gradually increased and then decreased force, the raw EMG signals were filtered by the filter box, after which the signal was sent to the PC with an analog to digital (AD) board. Using Matlab software (R2015a, MathWorks, MA, USA), the integrated sEMG signal was fitted further. On the other hand, the tactile feedback data were recorded and sent to the PC synchronously through the 1394 port. Figure 10 shows the results of processed sEMG signals with the fitting method and the raw sEMG signal, including the muscle relax status and three statuses with different tactile feedback were added. After comparing the force exerted by the Phantom Premium and the relative sEMG signals, we come to the conclusion that with the increasing of the exerted force, the sEMG signals also increased. The tactile feedback is beneficial for patients to achieve goal directed tasks by sensing the position errors between the affected and unaffected limbs.

### 4.2. Performance Evaluation of the Bilateral Training System

Using the proposed bilateral training system, the patient can retain his/her experience of motion in the unaffected limb, and the motor function of the affected limb can recover under the guidance of the experience, especially for hemiparesis stroke patients. It is necessary to verify the effectiveness of the proposed training system, including the recovery effect after adding the tactile and visual feedback, force assisting with the exoskeleton and training model. 

In our study, three subjects with physical fitness were involved for verifying the effectiveness of the proposed training system, including a female aged 26 and two males aged 25 and 27. The exoskeleton device is easy worn by patients and a caregiver is needed to operate the system. It should be noted that the exoskeleton can provide from relatively high joint impedance to near-zero impedance. In this experiment, the generating impedance from exoskeleton was set to a near-zero impedance, because the physically fit subjects were selected, as well as to verify the performance that the exoskeleton can provide at a near-zero impedance.

The threshold *H* is an important value for obtaining an optimal training experience. During the experiment, we set the threshold *H* as five degrees, which can be adjusted for the different statuses of patients, that is, when the difference angle between two limbs is less than five degrees, the target will change its position. Here, we mainly aim to test the effectiveness of the system during the training; the threshold should be carefully chosen according to the patients’ status.

**Figure 11:**
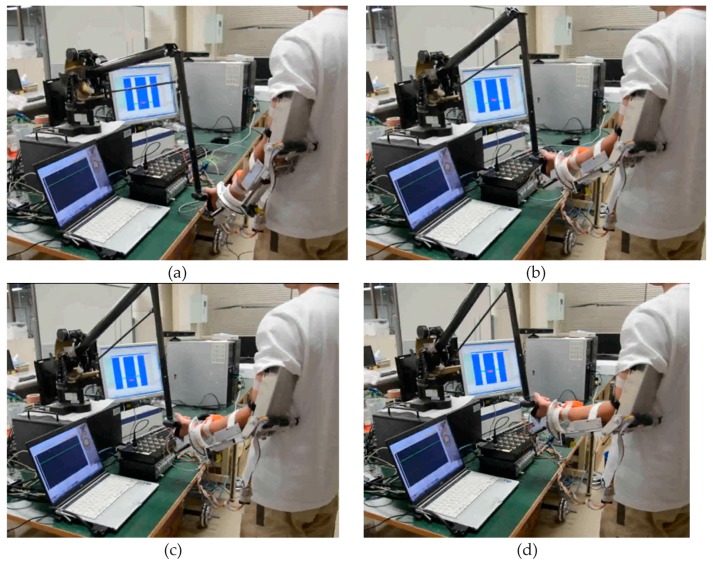
A subject is performing the bilateral training. (**a**) Phase 1, the affected arm operates the handle of the Phantom while synchronously moving together with the unaffected limb to the target position. (**b**) Phase 2, the two arms reach the directed position. (**c**) Phase 3, the target object will move to a random position within the moving ability of patients. (**d**) Phase 4, the two arms move to the renewed position to undergo the next round of training.

Figure 11 shows a subject performing the training while watching the graphic interface. The training can provide a goal-oriented task, where the target position is represented with a red square. During the training, the affected arm (left arm) operates the handle of the Phantom while synchronously moving together with the unaffected limb (right limb) to the target position, as shown in Figure 11a,b. After the two arms reach the directed position, the target object will move to a random position within the moving ability of patients, as shown in Figure 11c. Then, the two arms move to the renewed position to achieve the next round of training, as shown in Figure 11d. Each subject underwent five groups of experiments, and each group contained two contrastive experiments including one without tactile feedback and one with tactile feedback. The concrete information that the force provided by the Phantom was not told to the subject in order to ensure the accuracy of the contrastive experiment. Each subject was requested to perform 20 tasks, and the speed and duration were not limited. 

## 5. Results and Discussion

**Figure 12:**
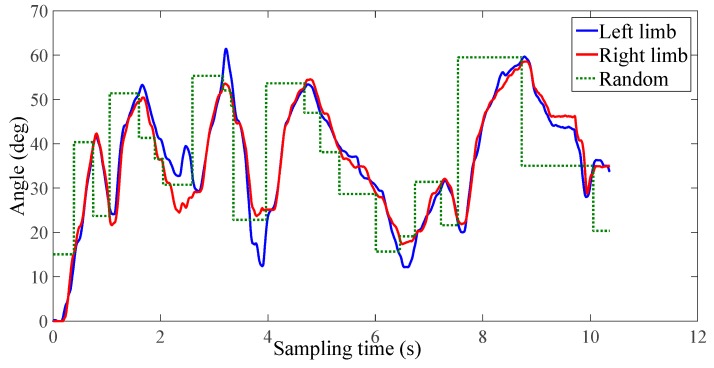
No tactile feedback (the third group, subject one).

**Figure 13:**
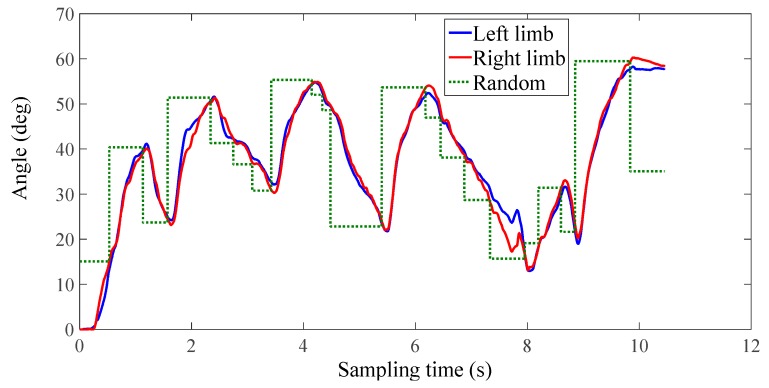
With tactile feedback (the third group, subject one).

**Figure 14:**
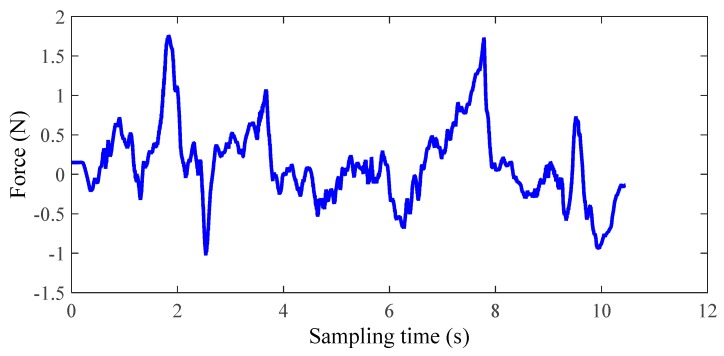
Tactile feedback from the Phantom (the third group, subject one).

The experimental results of the third group of subject one are shown in Figure 12, Figure 13 and Figure 14. In this study, only healthy subjects were involved for evaluating the system on three aspects. Firstly, the tactile feedback from the haptic device can provide task-oriented force feedback according to the training model. Secondly, the haptic feedback can help to speed up the learning process. Thirdly, the exoskeleton can provide a near-zero impedance, which is a precondition for providing an assist-as-needed force. In Figure 14, it can be seen that the magnitude and direction of tactile feedback changes along with the relative position between the two limbs, which is accordance with our conditions. Figure 12 and Figure 13 illustrate one contrastive experimental result. It can obviously be found that the error between the two limbs is smaller after adding the tactile feedback, that is, the motor function of the affected limb can recover well under the guidance of the experience from the unaffected limb with feedback.

The other results were appraised further with the squared error, as in Equation (16).

(16) $$\delta =\sum_{i=1}^{N}\frac{{\left(x_{affected}(i)-x_{unaffected}(i)\right)}^{2}}{N}$$

where *x_affected_* (*i*) represents the trajectory of the affected limb and *x_unaffected_* (*i*) is the trajectory of the unaffected limb. *N* is the sampling number, which is relative to the time and sampling frequency.

**Figure 15:**
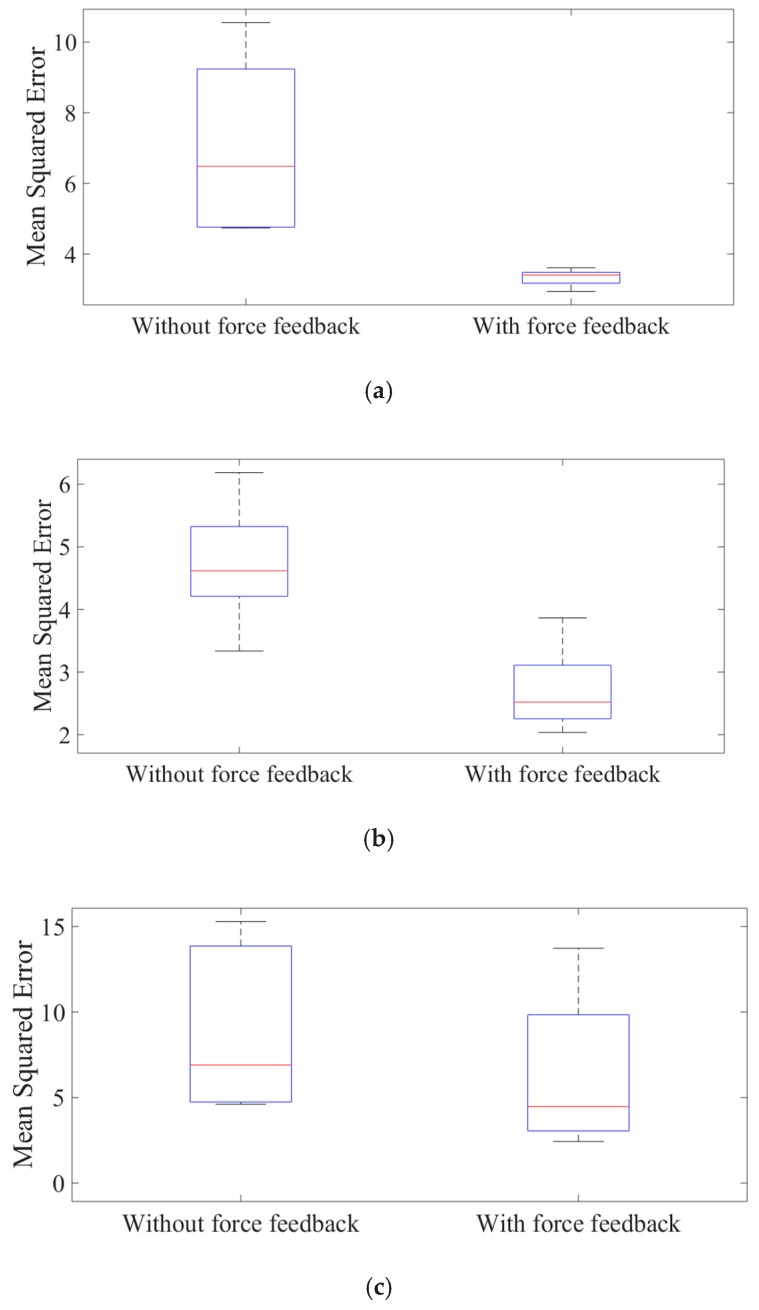
The mean squared error of subject one. (**a**) Subject one (female, age of 26); (**b**) subject two (male, age of 25); (**c**) subject three (male, age of 27).

After analyzing the statistical data shown in Figure 15, it can be concluded that the feedback can help to speed up the learning process. In detail, the mean squared error of the subject without force feedback is 7.0, 4.7, and 9.0 for three subjects, respectively, while the mean squared error with force feedback is 3.3, 2.7, and 6.9, respectively.

Moreover, during the experimental process, some phenomenon for different individuals should be noticed. Firstly, it may be caused by the instinct of humans; for some subjects, the limb will involuntarily resist the force coming from the haptic device. Secondly, for some subjects, they are inclined to use the unaffected limb to assist their affected limbs. For instance, in the case of upward motion, the subject would consciously make their unaffected limbs higher than the affected arm, thus the haptic device will provide an upward force. Thirdly, more force assisting than necessary is sometimes delivered, which results in an excessive reliance on exoskeletons. 

## 6. Conclusions

In this paper, the bilateral training system on the sagittal plane for elbow joint recovery is proposed. Patients can obtain the neural training with the cooperative movement between the affected and unaffected arm. This system is designed according to the neuro-rehabilitation theory. The motor function can be recovered with cooperative training between the affected limb and unaffected limb via both tactile and virtual feedback. A commercial haptic device (Phantom Premium) can effectively provide tactile feedback on errors and how to correct them. A contract test was carried out between the training with or without tactile feedback. By analyzing the data recorded from three healthy subjects, the tactile feedback was proven to be effective. Nero-zero impedance was set to the exoskeleton, which is worn on the affected limb to provide assisting force, and near-zero impedance was set to prove that the weight of the exoskeleton device will not influence the power assisting function.

In the future, more training models will be designed for other independent joints. More training parameters such as the training duration and speed will be considered. To obtain a comparable evaluation, the motor behaviors of healthy subjects and stroke patients will be assessed.
